# Eosinophilic Liver Disease Mimicking Hepatic Metastases

**DOI:** 10.5334/jbr-btr.1235

**Published:** 2017-03-29

**Authors:** Vinciane Vercruysse, Lieven Van Hoe

**Affiliations:** 1 Departments of Radiology, OLV-Hospital, Moorselbaan 164, 9300 Aalst, BE

**Keywords:** Eosinophilia, liver, metastases

## Abstract

In rare cases of hypereosinophilic syndrome, nodular lesions of liver due to infiltration of eosinophilic granulocytes has been described. In such cases, a computed tomography of the abdomen could mimic a metastasizing disease while a spontaneous regression of the lesions can be expected. We will present such a case and discuss how this misdiagnosis can be avoided.

## Case Presentation

**Figure 1:**
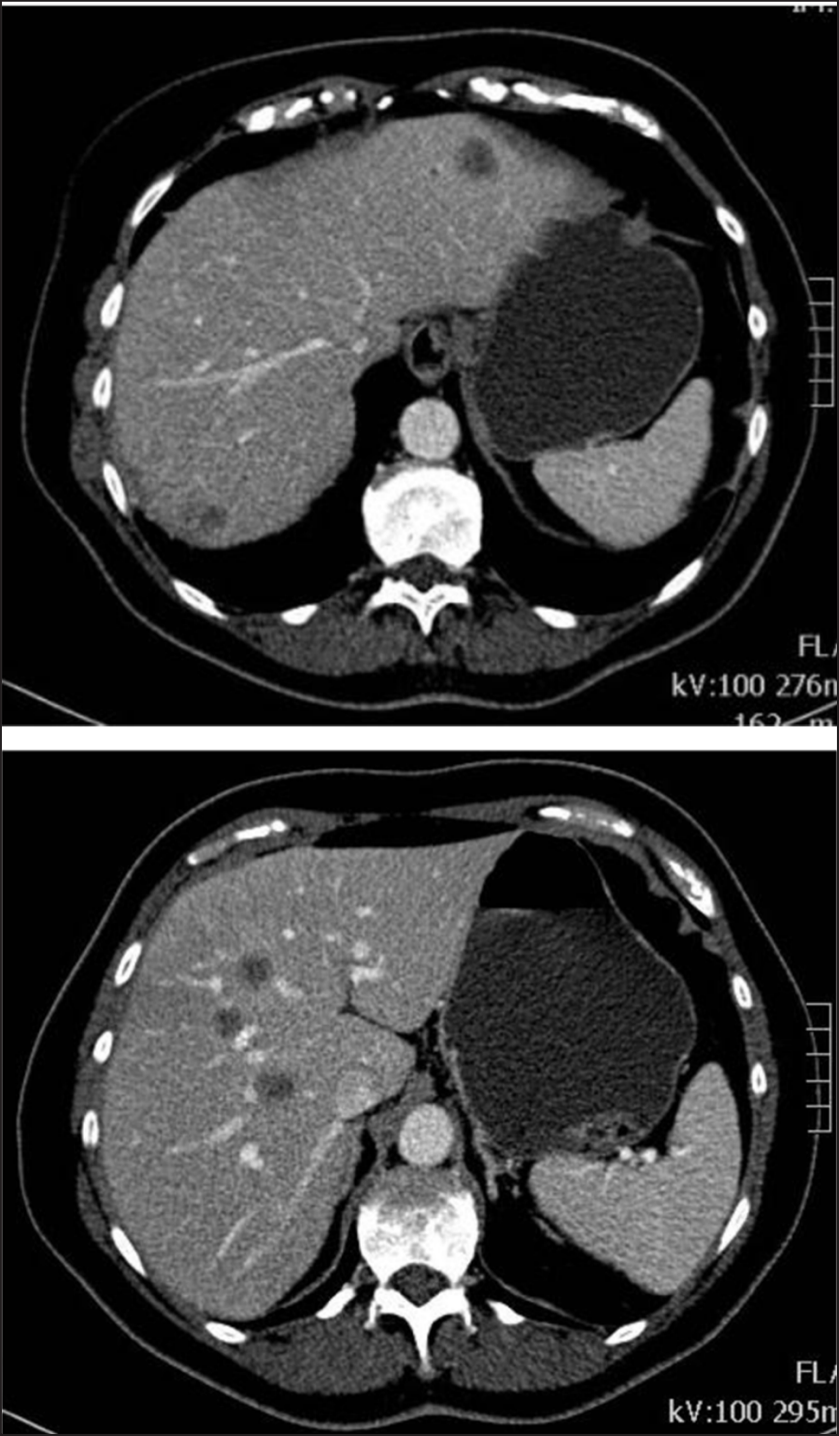
Contrast-enhanced CT abdomen in the portal venous phase: multiple focal low-attenuation nodular lesions in the liver.

**Figure 2:**
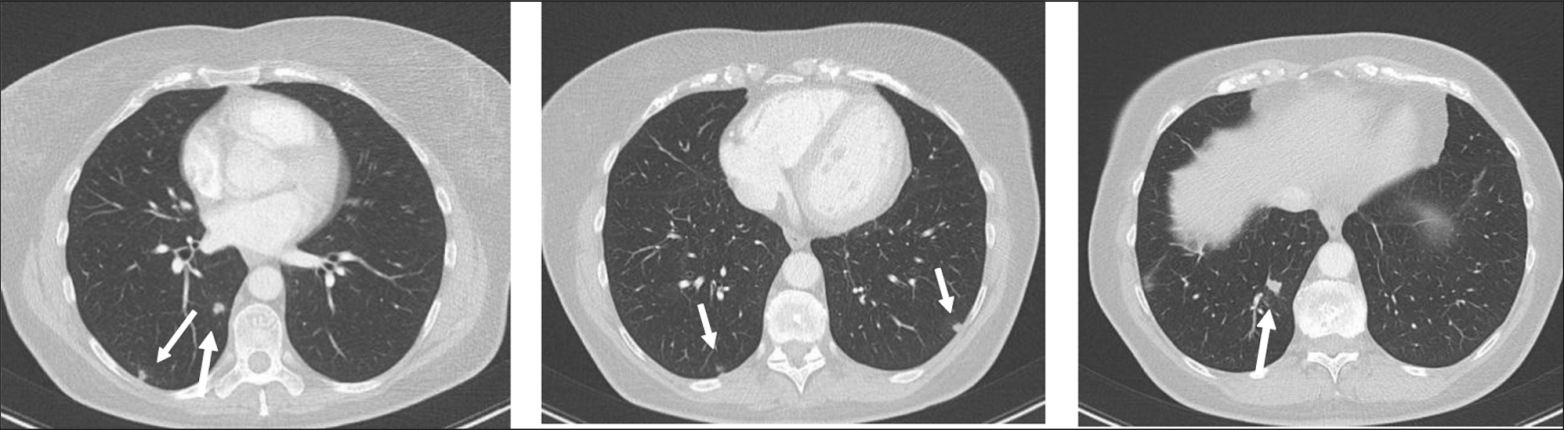
CT thorax: multiple small nodular lung lesions (arrows).

A 59-year-old woman consulted her general practitioner for a two-week episode of widespread itchiness, enlarged cervical nodes on the left side and right upper quadrant pain. She felt feverish. Her medical history included appendectomy and hysterectomy. Medication consisted of benzodiazepines. Initial laboratory examination was normal except for leukocytosis of 1500/µl (normal range: 400–1000/µl) and eosinophilia of 4400 cells/µl (normal range: 0–400 cells/µl). Her general practitioner requested abdominal computed tomography (CT). It showed multiple focal low-attenuation lesions in the liver (Figure 1), considered suspicious for liver metastases, and a small nonspecific lesion in the left kidney. Additionally, a chest CT was performed and showed small nodular lesions, potentially indicating lung metastases (Figure 2).

The patient was referred to the urology department, because the lesion in the left kidney could possibly represent the primary tumor. However, this preliminary hypothesis was rejected, as the renal lesion did not show typical features of renal cell carcinoma on a dedicated triphasic renal CT. The liver and lung manifestations were therefore considered potentially as a metastatic disease of unknown etiology.

The patient was referred to the oncology department for further investigation. Fluorodeoxyglucose positron emission tomography was performed and didn’t show any pathological tracer accumulation in the lung, liver or kidney. Laboratory tests showed (besides eosinophilia of now 540 cells/µl), normal CA 125, CA 15.3, CA 19.9, CEA and NSE tumor markers. As a next step, an ultrasound-guided liver biopsy was performed. The pathology report described a dense infiltrate of eosinophilic granulocytes without any malignant epithelial cell. Because clinicians still considered malignancy very likely, a second liver biopsy was requested and showed normal liver parenchyma with rare lymphocytes and eosinophils, again without any signs of malignancy. The hypothetic diagnosis of hypereosinophilic syndrome was assumed. Because of the spontaneous symptomatic improvement and the rather mild peripheral eosinophilia, a “wait and see” attitude was adopted, and the patient was discharged with close follow up. After two months, a follow-up CT of the thorax and the abdomen showed complete regression of the lung lesions and significant volume decrease of the liver lesions. Laboratory results showed peripheral eosinophils within the normal range.

## Discussion

Hypereosinophilia in the peripheral blood is defined as an absolute eosinophil count of more than 1500 cells/µl on two examinations separated in time by at least one month and/or pathologic confirmation of tissue hypereosinophilia [4]. In our case, there was only one laboratory examination that showed eosinophilia above 1500 cells/µl (4400 cells/µl); the pathologic examination of the liver biopsy confirmed the diagnosis of hypereosinophilia. Hypereosinophilic syndrome is a condition characterized by prolonged peripheral blood eosinophilia in association with eosinophil-mediated organ damage or dysfunction, provided other potential causes for the damage (parasitic infection, drug hypersensitivity, allergic disease, collagen vascular disease and neoplastic disease) have been excluded [3,4]. Every organ can be involved; in our case the CT scan revealed nodular lesions in liver and lungs. Those lesions indicate an active disease condition, justifying therapy with corticosteroids in case of lack of spontaneous regression.

In this case, the diagnosis was put on the wrong track by misinterpreting the CT scans, unfortunately, leading to an anxious patient and a second (unnecessary) liver biopsy. The challenge in this case is to distinguish eosinophilic lesions in the liver from metastatic liver disease, which is especially important in patients with a known primary tumor. The different radiographic characteristics of eosinophilic liver disease and malignant nodules are well described in several reports. On CT, in the portal venous phase, the eosinophilic lesions and malignant nodules are both low-attenuation lesions. Eosinophilic lesions are characterized by multifocal, small (< 2 cm) oval or round lesions with irregular margins, whereas malignant nodules are usually (but not necessarily) significantly larger and well-circumscribed [1]. More helpful than morphologic features is correlation with lab testing and follow-up. Follow-up CT typically shows regression or disappearance of the eosinophilic lesions, whereas malignant nodules generally progress [1]. On MRI, the two entities appear quite similar and diffusion-weighted imaging may not be helpful to differentiate these hepatic lesions [3,5].

## Conclusion

Eosinophilic disease is a rare condition that may mimic liver and lung metastases. The radiologist should be aware of the existence of this condition and add it to the list of rare differential diagnostic considerations, particularly in patients with peripheral eosinophilia and normal tumor markers. CT findings in favor of eosinophilic lesions include multifocal, small (<2 cm) oval or round lesions with irregular margins. Imaging-guided biopsy together with correct interpretation of imaging findings and follow-up allow correct diagnosis. Especially in patients with concurrent neoplastic disease, this may avoid misdiagnosis and even unnecessary chemotherapy [1,2].
